# Statistical analysis plan for the replacing protein via enteral nutrition in a stepwise approach in critically ill patients (REPLENISH) randomized clinical trial

**DOI:** 10.1186/s13063-024-08105-w

**Published:** 2024-05-02

**Authors:** Yaseen M Arabi, Hasan M. Al-Dorzi, Omar Aldibaasi, Musharaf Sadat, Jesna Jose, Dina Muharib, Haifa Algethamy, Abdulrahman A. Al-Fares, Fahad Al-Hameed, Ahmed Mady, Ayman Kharaba, Ali Al Bshabshe, Khalid Maghrabi, Khalid AlGhamdi, Ghulam Rasool, Adnan AlGhamdi, Ghaleb. A Almekhlafi, Jamal Chalabi, Haifaa Ibrahim AlHumedi, Maram Hasan Sakkijha, Norah Khalid Alamrey, Amjad Sami Alaskar, Rabeah Hamad Alhutail, Kaouthar Sifaoui, Rakan Alqahtani, Ahmad S. Qureshi, Mohammed Moneer Hejazi, Hatim Arishi, Samah AlQahtani, Amro Mohamed Ghazi, Saleh T. Baaziz, Abeer Othman Azhar, Sara Fahad Alabbas, Mohammed AlAqeely, Ohoud AlOrabi, Aliaa Al-Mutawa, Maha AlOtaibi, Madiha Fawazy Elghannam, Mohammed Almaani, Sarah Fadel Buabbas, Wadiah Alawi M. Alfilfil, Mohammed S. Alshahrani, Joel Starkopf, Jean-Charles Preiser, Anders Perner, Jumana Hani AlMubarak, Wafa Mansoor Hazem, Talal Albrahim, Abdulaziz Al-Dawood, and the Saudi Critical Care Trials Group, Amal Almatroud, Brintha Naidu, Vicki Burrow, Salha Al Zayer, Haseena Banu Khan, Afonso Varela, Hatim Arishi, Mohammed Moneer Hejazi, Mohamed Ali Alodat, Rayan Alshayeh, AbdulRehman AlHarthi, Naif Al Qahtani, Yasmeen Ayed AlHejiely, Mada Muzhir AlZahrani, Mohammed Haddad Lhmdi, Nouf AlBakhiet, Katrina Baguisa, Huda Mhawisg, Haifa Alghethamy, Liyakat Khan, Moataz Gabr, Shehla Nuzhat, Ohoud AlOrabi, Raghad Malabari, Kholoud Shobragi, Shaymaa Asaas, Madiha Fawazy Elghannam, Beverly Bcuizon, Bander AlAnezi, Christine Joy Anaud, Munir AlDammad, Yahia Otaif, Osama Hakami, Arwa AlHusseini, Shahinaz Bashir, Lama Hefni, Samahar Alamoudi, Milyn L. Ansing, Sawsan Albalawi, Manar Alahmadi, Mohammed AlHumaid, Samar Talal Nouri, Rozeena Huma, Khawla Farhan, Mohamed Hussein, Olfa Baji, Abdulrehman Alerw, Khloud Johani, Monera AlEnezi, Ismail Boudrar, Rabiah Atiq, Maali Junid, Maram Yusef, Ahmed Quadri, Khalid Idrees, Mona Bin Mabkoot, Wadiah Alawi Alfilfil, AbdulRehman Fahad Alkraidees, Laila Perlas Asonto

**Affiliations:** 1 Intensive Care Department, King Abdulaziz Medical City, Ministry of National Guard Health Affairs, King Abdullah International Medical Research Center, ICU, College of Medicine, King Saud Bin Abdulaziz University for Health Sciences, Riyadh, Saudi Arabia; 2 King Abdullah International Medical Research Center, King Saud Bin Abdulaziz University for Health Sciences, Riyadh, Saudi Arabia; 3 Intensive Care Department, King Saud Medical City, Riyadh, Saudi Arabia; 4 Department of Anesthesia and Critical Care, King Abdulaziz University, King Abdulaziz University Hospital, Jeddah, Saudi Arabia; 5 Department of Anesthesia, Critical Care Medicine and Pain Medicine, Al-Amiri Hospital, Ministry of Health, Kuwait City, Kuwait; 6 Intensive Care Department, King Abdulaziz Medical City, Ministry of National Guard Health Affairs, King Abdullah International Medical Research Center, King Saud Bin Abdulaziz University for Health Sciences, Jeddah, Saudi Arabia; 7 Department of Anesthesiology and Surgical Intensive Care, Tanta University Hospitals, Tanta, Egypt; 8 Pulmonary & Critical Care Departments, King Fahad Hospital, Critical Care Units- Madinah Region, Ministry of Health, Madinah, Saudi Arabia; 9 Department of Critical Care Medicine, Aseer Central Hospital, King Khalid University, Abha, Saudi Arabia; 10 Internal Medicine, Critical Care, King Faisal Specialist Hospital and Research Centre, Riyadh, Saudi Arabia; 11 Internal Medicine, Critical Care, King Faisal Specialist Hospital and Research Centre, Jeddah, Saudi Arabia; 12 Department of Intensive Care Services, Prince Sultan Military Medical City, Riyadh, Saudi Arabia; 13 Intensive Care Department, King Salman Bin Abdulaziz Medical City, Madinah, Saudi Arabia; 14 Intensive Care Department, King Abdulaziz Medical City, King Abdullah International Medical Research Center, AlAhsa Ministry of National Guard Health Affairs, King Saud Bin Abdulaziz University for Health Sciences, Riyadh, Saudi Arabia; 15 Department of Critical Care, College of Medicine, King Saud University, Riyadh, Saudi Arabia; 16 Intensive Care Department, Prince Mohammed Bin Abdulaziz Hospital, Ministry of National Guard Health Affairs, King Abdullah International Medical Research Center, King Saud Bin Abdulaziz University for Health Sciences, Madinah, Saudi Arabia; 17 Adult Critical Care Services, King Fahad Medical City, Riyadh, Saudi Arabia; 18 Department of Anesthesia, Critical Care and Pain Medicine, Jaber Al-Ahmed Al-Sabah Hospital, Kuwait City, Kuwait; 19 Department of Intensive Care, Prince Mohammed Bin Abdulaziz Hospital, Riyadh, Saudi Arabia; 20 Department of Emergency and Critical Care, King Fahad Hospital of the University, Imam Abdulrahman Bin Faisal University, Al Khobar, Saudi Arabia; 21 Clinic of Anaesthesiology and Intensive Care, University of Tartu, Tartu University Hospital, Tartu, Estonia; 22 Department of Intensive Care, Erasme University Hospital, Brussels, Belgium; 23 Department of Intensive Care, Rigshospitalet, University of Copenhagen, Copenhagen, Denmark

**Keywords:** Critical illness, Protein, Statistical analysis plan, Randomized clinical trial, Nutrition

## Abstract

**Background:**

The optimal amount and timing of protein intake in critically ill patients are unknown. REPLENISH (Replacing Protein via Enteral Nutrition in a Stepwise Approach in Critically Ill Patients) trial evaluates whether supplemental enteral protein added to standard enteral nutrition to achieve a high amount of enteral protein given from ICU day five until ICU discharge or ICU day 90 as compared to no supplemental enteral protein to achieve a moderate amount of enteral protein would reduce all-cause 90-day mortality in adult critically ill mechanically ventilated patients.

**Methods:**

In this multicenter randomized trial, critically ill patients will be randomized to receive supplemental enteral protein (1.2 g/kg/day) added to standard enteral nutrition to achieve a high amount of enteral protein (range of 2–2.4 g/kg/day) or no supplemental enteral protein to achieve a moderate amount of enteral protein (0.8–1.2 g/kg/day). The primary outcome is 90-day all-cause mortality; other outcomes include functional and health-related quality-of-life assessments at 90 days. The study sample size of 2502 patients will have 80% power to detect a 5% absolute risk reduction in 90-day mortality from 30 to 25%. Consistent with international guidelines, this statistical analysis plan specifies the methods for evaluating primary and secondary outcomes and subgroups. Applying this statistical analysis plan to the REPLENISH trial will facilitate unbiased analyses of clinical data.

**Conclusion:**

Ethics approval was obtained from the institutional review board, Ministry of National Guard Health Affairs, Riyadh, Saudi Arabia (RC19/414/R). Approvals were also obtained from the institutional review boards of each participating institution. Our findings will be disseminated in an international peer-reviewed journal and presented at relevant conferences and meetings.

**Trial registration:**

ClinicalTrials.gov, NCT04475666. Registered on July 17, 2020.

**Supplementary Information:**

The online version contains supplementary material available at 10.1186/s13063-024-08105-w.

## Background

The optimal amount and timing of protein intake in critically ill patients are unknown. Based on the limited existing data, different clinical practice guidelines recommended a wide range of doses of proteins for critically ill patients, from 1.2 to 2.5 g/kg/day [1].

Given the uncertainty regarding the optimal dose of protein intake in critically ill patients [2–10] and the limited data from randomized clinical trials (RCTs), we are conducting the Replacing Protein via Enteral Nutrition in a Stepwise Approach in Critically Ill Patients (REPLENISH) trial. This trial is an open-label, multicenter RCT that evaluates whether supplemental enteral protein (1.2 g/kg/day) added to standard enteral nutrition to achieve a high amount of enteral protein (range 2–2.4 g/kg/day) given from ICU day 5 until ICU discharge up to ICU day 90 as compared with no supplemental enteral protein to achieve a moderate amount of enteral protein (0.8–1.2 g/kg/day) will reduce all-cause 90-day mortality in adult critically ill patients.

This manuscript describes the statistical analysis plan (SAP) for the REPLENISH trial, in compliance with the “Statistical Principles for Clinical Trials E9” report and “Structure and Content of Clinical Study Report E3” [11, 12]. This statistical analysis plan identifies the procedures to be applied to the primary and secondary analyses once trial data validation is complete. All analyses were prospectively defined, and the SAP was finalized by the Principal Investigator and the Steering Committee members before the final analysis. Participant recruitment is expected to be completed in April 2025. The final study report will follow the Consolidated Standards of Reporting Trials (CONSORT) 2010 guidelines for reporting RCTs [13, 14].

## Methods

### Study design

The REPLENISH trial is an open-label, parallel-group, multicenter superiority RCT currently enrolling patients from 17 hospitals in 2 countries. The study will be conducted according to the principles of the latest version of Good Clinical Practice and in accordance with all relevant local ethical, regulatory, and legal requirements. In Saudi Arabia, the trial is approved by the Institutional Review Board (IRB) of Ministry of National Guard Health Affairs, Riyadh, Saudi Arabia (Protocol number RC19/414R). In other sites, the study has been approved by their respective IRBs. The trial is registered in ClinicalTrials.gov (NCT04475666), and the study protocol has been previously published [15].

According to local regulations, the patients will be consented through their surrogate decision-maker. Some sites have approval for using a deferred consent model in case the consent was not taken a priori. Medical, surgical, and trauma ICU patients will be screened on day 4 of ICU stay up to the morning of day 5. Adult mechanically ventilated patients (≥ 18 years old) on enteral nutrition who are unlikely to be discharged from the ICU on the following day will be included in the study. Exclusion criteria have been described in a previously published protocol [15]. Eligible patients will be randomized to the supplemental protein group (2–2.4 g/kg/day) or the control group (0.8–1.2 g/kg/day). Protein requirement will be calculated based on actual body weight for patients with a body mass index (BMI) < 30 kg/m^2^. For patients with a BMI ≥ 30 kg/m^2^, we will calculate the adjusted body weight as follows: adjusted body weight (in kg) = ideal body weight + 0.4 * (actual weight – ideal body weight). The ideal body weight will be calculated as follows: for men: 50 + (0.91 × [height in centimeters − 152.4] and for women: 45.5 + (0.91 × [height in centimeters − 152.4]). Randomization is achieved through a secure web-based randomization system using permuted variable undisclosed block sizes. Randomization is stratified according to the trial site, the use of renal replacement therapy at the time of randomization and whether the patient is a suspected or confirmed case of COVID-19. The study intervention starts from ICU day 5 (midnight) and is continued till meeting any of the following criteria: death, ICU discharge or day 90 in ICU, premature stopping of feeding due to brain death or palliative care plan, or initiation and tolerance of full oral feeding for more than 24 h, whichever comes first. Patients are followed up daily until day 90 if in the ICU or until ICU discharge and then at day 90.

### Study population

**Fig. 1:**
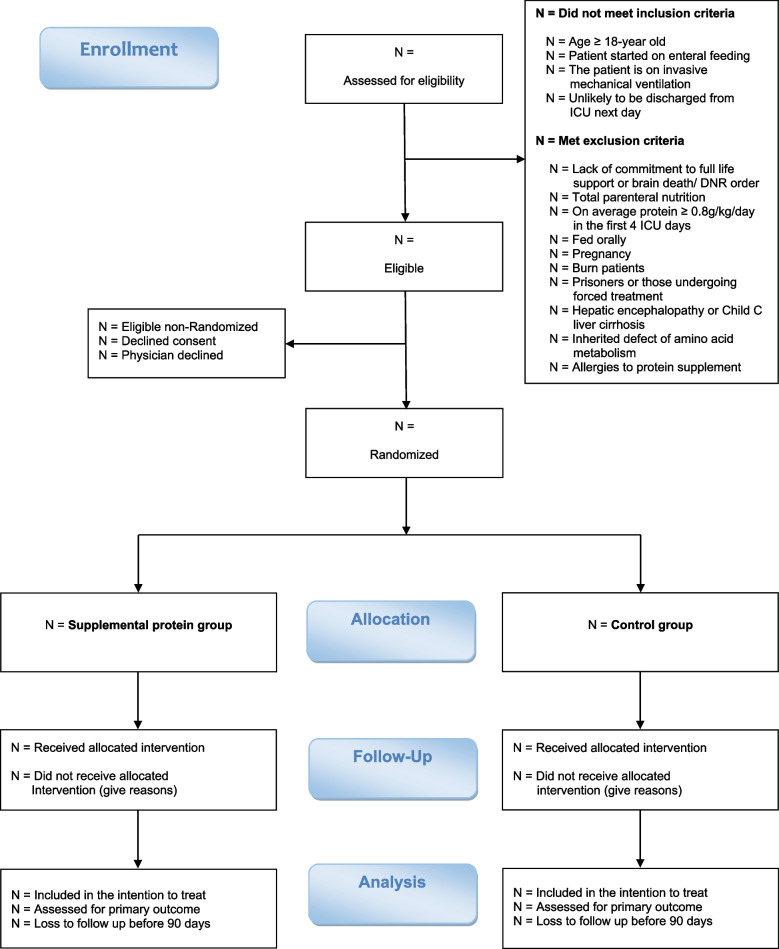
Consort flow diagram

The flow of patients through the study will be displayed in a CONSORT diagram (Fig. 1). We will report the number of patients screened, met inclusion or exclusion criteria, were enrolled, and were eligible but not enrolled. Reasons for the exclusion of non-included patients will be reported. We will also report the number of patients who were randomized to each group, received the allocated interventions, and had the primary outcome data.

The primary analyses will be performed on the modified intention-to-treat population of all enrolled patients, whether or not they received the allocated interventions. Post-enrollment exclusion from the modified intention-to-treat population will be limited to the cessation of study procedures due to withdrawal of consent. The data of these patients would only remain in the analyzed dataset if the patient or surrogate decision-maker consented to use trial data. Patients will also be excluded post-enrollment from the modified intention-to-treat population if the eligibility criteria were found not to have been met and study interventions were not started [16]. Censoring dates will only occur in case of “real” loss to follow-up (i.e., discharged patients with no information beyond some point). In that case, the censoring date will be the last day of contact or the date of hospital discharge if no other information is available.

## Data

### Baseline characteristics

We will present baseline age, sex, admission category (medical, postoperative (non-trauma) and trauma (postoperative and non-operative)), Acute Physiology and Chronic Health Evaluation (APACHE) II score, and chronic health points (defined as per the APACHE II system) [17]. Baseline data (pre-randomization ICU day 4) will include morning blood glucose, sedatives and neuromuscular blockers infusions, and systemic corticosteroid use. The Sequential Organ Failure Assessment (SOFA) score will also be reported on day 4. We will also present the pre-morbid functional assessment for sarcopenia using the SARC-F (Strength, Assistance with walking, Rising from a chair, Climbing stairs, and Falls) score [18] (Supplementary Appendix 1-Table 1).

**Table 1:** Summary of the analysis plan

Variables	Test
Baseline characteristics	No statistical comparisons
Intervention and co-interventions	Chi-square, Fisher’s exact test, Mann–Whitney *U* test, *t*-test as applicable For serial values: generalized linear mixed effect models
Primary outcome	1. Primary analysis: generalized mixed effects model with adjustment to stratification variables. Reporting of risk difference and relative risk 2. Secondary analyses: a. Chi-square or Fisher’s exact test b. Sensitivity analyses using generalized mixed effects model with adjustment to stratification variables and multiple imputations c. Cox proportional analysis d. Adjusted Cox proportional analysis e. Kaplan–Meier curves
Secondary outcomes	1. Categorical variables: generalized mixed effects model with adjustment to stratification variables. Reporting of risk difference and relative risk 2. Continuous variables: linear regression model and van Elteren test as appropriate. The results will be reported as medians and mean differences and beta coefficients with 95% CI. For HRQoL: sensitivity analysis using multiple imputations 3. Multiplicity adjustment by false discovery rate (FDR)
Safety outcomes	Generalized mixed effects model with adjustment to stratification variables and reporting of risk difference and relative risk.
Subgroup analyses	Generalized mixed effects model with adjustment to stratification variables and reporting of risk difference, relative risk, tests of interaction and FDR.

### Intervention and co-interventions

We will report the estimated and administered energy and protein requirements for each group. Energy intake will include energy from enteral nutrition (including protein in the primary formula) and intravenous dextrose, citrate, propofol, and parenteral nutrition (if any). We will report total energy without and with the energy from supplemental protein. Protein intake includes the protein from the primary formula in both groups, the supplemental protein in the supplemental protein group and parenteral protein (if any). Energy and protein will be reported as kcal/kg based on actual body weight for patients with a BMI < 30 kg/m^2^ and adjusted body weight for those with a BMI ≥ 30 kg/m^2.^ To ensure that energy and protein intake data are collected for complete 24-h periods, nutrition data will not be included for the last day of intervention if the duration of intervention on that day is less than 24 h.

We will report daily blood glucose, serum creatinine, and urine output by group. We will compare serial weights and the highest mobility level during the ICU stay [19]. We will also compare serial prealbumin, albumin, ammonia, blood urea nitrogen, 24-h urine for urinary urea nitrogen, lowest potassium level, lowest magnesium level and lowest phosphate level as well as the levels of aspartate transaminase, alanine aminotransferase, and international normalized ratio. We will report the average daily insulin dose during the ICU stay. We will also report the use of corticosteroids and statins in the two groups during the study period (Supplementary Appendix 1-Table 2).

#### Study outcomes

##### Primary outcome

Ninety-day all-cause mortality is defined as death by day 90 from the ICU admission date.

##### Secondary outcomes

- Days alive at day 90 without life support, which will be calculated as the total number of days alive and free of vasopressor use, invasive mechanical ventilation and renal replacement therapy within 90 days after randomization. As a supplementary analysis, we will report the components of this composite outcome: vasopressor-free days, invasive mechanical ventilation-free days, and renal replacement-free days. Patients who die during the 90-day follow-up will be assigned 0 free days.
- Days alive and out of the hospital at day 90 will be calculated as the number of days from alive hospital discharge to day 90. Patients who die during the 90-day follow-up will be assigned 0 free days.
- Bacteremia defined as positive blood cultures, excluding those considered contaminant organisms, until 2 days post ICU discharge.
- New or progression of skin sacral pressure ulcers in ICU (http://www.npuap.org/resources/educational-and-clinical-resources/npuap-pressure-ulcer-stagescategories/), using the definitions of the National Pressure Ulcer Advisory Panel, which include stage I: non-blanchable erythema, stage II: partial-thickness skin loss, stage III: full-thickness skin loss, and stage IV: full-thickness tissue loss.
- Functional assessment using SARC-F (strength, assistance with walking, rising from a chair, climbing stairs, and falls) score for sarcopenia [18] at day 90.
- EuroQoL 5-Dimension 5-Level (EQ-5D-5L) [20] index value and EQ visual analog scale (EQ-VAS) at day 90. The EQ-5D-5L has five dimensions (mobility, self-care, usual activities, pain or discomfort, and anxiety/depression) with five levels of severity (no problems, slight problems, moderate problems, severe problems, extreme problems). A higher score indicates a worse condition [20]. The scores of each patient will be first converted into a single index value. The EQ-5D-5L index value will be calculated using the Kingdom of Saudi Arabia value sets if they become available at the time of analysis; otherwise, we will use the United States EQ-5D-5L value sets [21]. Patients who die by the 90-day follow-up will be assigned 0 values in both index value and EQ-VAS. Data will also be presented for survivors only.
- *Safety outcomes* are classified into major and minor safety outcomes.

Major safety outcomes include

- ➢ New episode of stage 2 or higher of acute kidney injury by KDIGO (Kidney Disease Improving Global Outcomes) criteria [22] after enrollment. This is defined as a new initiation of renal replacement therapy after randomization, an increase in creatinine by ≥ 2.0-folds compared to the baseline creatinine (the lowest available value before randomization) or urine output < 0.5 ml/kg/h on any given day post-randomization.
- ➢ Newly confirmed pneumonia according to the modified CDC criteria [23].
- ➢ Grade IV acute gastrointestinal injury [24] including any bowel ischemia with necrosis, clinically important gastrointestinal bleeding, Ogilvie’s syndrome, and abdominal compartment syndrome.

Minor safety outcomes include

- ➢ Feeding intolerance defined as vomiting or large gastric residual volume (≥ 500 ml/24 h) on a single calendar day.
- ➢ Diarrhea defined as having three or more loose or liquid stools per day with a stool weight > 200–250 g/day (or > 250 ml/day) [24].
- ➢ Refeeding syndrome defined as a fall in serum phosphate below 0.65 mmol/L within 72 h of starting nutritional support and the drop being > 0.16 mmol/L from a previously recorded reading during ICU stay [25, 26].

#### Statistical analysis plan

A. *General concepts*: Categorical variables will be reported as numbers and frequencies. Continuous variables will be reported as means and standard deviations or medians and interquartile ranges, as judged appropriate by normality testing. Details of missing data (numbers and proportions) will be provided. Categorical variables will be compared using the Chi-square test. Continuous variables will be compared using the Student’s *t*-test or the Wilcoxon–Mann–Whitney test, as judged appropriate by normality testing. For serial measurements, we will test the change over time and the difference between the two groups over time using generalized linear mixed-effect models. These will be graphically represented. Unless otherwise specified, tests will be two-sided at 5% significance level. Analyses will be performed using the SAS software version 9.1.3 or higher (SAS Institute, Cary, NC, USA).
B. *Sample size*: The study sample size of 2502 patients will have 80% power to detect a 5% absolute risk reduction in 90-day mortality from 30 to 25% [15].
C. *Multiplicity*: We will use the false discovery rate (FDR) to adjust for multiple testing for secondary outcomes and subgroup analyses, each as a separate group, as described by Benjamini and Hochberg [27].
D. *Analysis of primary outcome*: The primary outcome will be compared between the two groups using a generalized mixed effects model with adjustment to stratification variables [28, 29]. This approach of adjusting primary analysis for stratification variables has been suggested to avoid an unnecessary loss of power [28]. Results will be reported as risk difference (RD) and relative risk (RR) with 95% confidence intervals (CI) derived from the multivariable model with the maximum likelihood method. We will perform a secondary analysis using the Chi-square or Fisher’s exact test. To address the missing primary outcomes (loss-to-follow-up), we will perform sensitivity analyses using a multiple imputations model, in which missing values of the primary outcome will be imputed using a fully conditional specification technique with 100 imputations. Primary outcome will be predicted based on age, sex, the trial site, the use of renal replacement therapy at the time of randomization, and whether the patient is a suspected or confirmed case of COVID-19. We will also use Cox proportional hazard model without and with adjustment to stratification variables as secondary analyses, censoring by the last follow-up date, and the results will be reported as hazard ratio (HR) and 95% CI (Supplementary Appendix 1-Table 3). The distributions of time to death will be compared using Kaplan–Meier survival curves and a log-rank test. Table 1 presents the summary of statistics that will be performed on the primary and secondary outcomes.
E. *Analysis of secondary outcomes*: Secondary categorical outcomes will also be compared in the modified intention-to-treat cohort using a generalized mixed effects model with adjustment to stratification variables. Results will be reported as RDs, RRs, and 95% CIs. Continuous data such as the EQ-5D-5L index value, EQ-VAS, and SARC-F score collected 90 days post-randomization will also be analyzed using a linear regression model and van Elteren test as appropriate with adjustment to stratification variables. The results will be reported as medians and mean differences and beta coefficients with 95% CIs. Because data on EQ-5D-5L index value and EQ-VAS could be missing for some patients, we will conduct sensitivity analysis using multiple imputations. (Supplementary Appendix 1-Tables 4 and 5)
F. *Protocol violations and serious adverse events*: Protocol violations and serious adverse events will be reported and compared between the two groups (Supplementary Appendix 1-Table 6).
G. *Subgroup analyses*: Subgroup analysis will be performed for the primary outcome in the subgroups determined at baseline (Supplementary Appendix 1-Table 7). Results will be reported using RRs and 95% CIs, and the multivariable logistic regression will be used to report the results of tests of interactions for these subgroups. We will evaluate the effect of the intervention within the following subpopulations:
  Medical versus postoperative versus trauma
  Admission diagnosis of sepsis versus no sepsis
  Vasopressor use at the time of enrollment versus none
  Acute kidney injury at enrollment (4 KDIGO groups: 0, 1, 2, 3)
  Liver injury defined as AST or ALT at enrollment > 3 upper normal limit or bilirubin > 1.5 upper normal limit, which is consistent with hepatotoxicity stage 2 and above, according to the Cancer Therapy Evaluation Program of the National Cancer Institute (NCI) of the National Institutes of Health [30].
  COVID-19 versus no COVID-19
  BMI of ≤ 30 or > 30 kg/m.^2^
  High nutritional risk defined as a NUTRIC score of 5–9 and low nutritional risk as a NUTRIC score of 0–4
  SARC-F score of < 4 or ≥ 4
  Day 4 SOFA stratified at a median value

H. *Interim analyses*: Two interim analyses are planned to be conducted when 33% and 67% of the sample size (2502 patients) are achieved. The study has two biostatisticians, one involved in study design and analysis and the other in generating a closed report with unblinded group data. The interim analysis will be conducted for the primary outcome and safety outcomes. We will consider a *p*-value of < 0.01 for safety and a *p*-value of < 0.001 for effectiveness as early stopping criteria. There will be no plans to terminate the trial for futility. One additional unplanned interim analysis was conducted (see Trial status below). We will use a group sequential α-spending function, calculated using the O'Brien–Fleming method, with two-sided symmetric bounds, and the final *p*-value will be considered at 0.0412, considering the three interim analyses (the two originally planned and the additional unplanned analysis).
I. *Final analysis*: The final analysis will be conducted after the data on the 90-day mortality is completed [31].

#### Sub-studies

A. *REPLENISH-COVID sub-study:* We will evaluate the effect of high versus moderate protein on the subgroup of suspected or confirmed COVID-19 patients at enrollment. Critically ill patients with COVID-19 are in a state of high inflammation, increased stress, and catabolism. Poor oral intake, which may last 5 to 10 days before admission, is also common due to frequent coughing and breathlessness, dry mouth, and loss of taste and smell [32]. Long stay in the ICU, especially for intubated and ventilated patients, contributes to further malnutrition, loss of skeletal muscle mass, and disability. Though early and adequate enteral nutrition would be thought to mitigate these challenges and prevent gastrointestinal dysfunction [33, 34], it has the potential for adverse reactions like abdominal distention, diarrhea, regurgitation, and overfeeding [35]. Thus, the proper timing of optimal nutrients needed to meet the energy and protein requirements in critically ill patients with COVID-19 is debatable [35]. We will conduct a subgroup analysis based on COVID-19 status at baseline and assess the effect of protein intake on outcome. We will conduct a similar analysis to that of the main trial. We will conduct a sensitivity analysis using imputation to account for missing primary outcomes, if needed. In this model, missing values of the primary outcome variable will be imputed using a fully conditional specification multiple imputation technique with 100 imputations. Primary outcome values will be predicted from age, sex, the trial site, and the use of renal replacement therapy at the time of randomization. Additional baseline laboratory tests, including ferritin, interleukin-6 (IL-6), lactate, and procalcitonin, if available, will be compared between the two groups. In suspected or confirmed COVID-19 patients, we will also compare the use of extracorporeal membrane oxygenation, inhaled nitric oxide, prone positioning, tracheostomy, intravenous immunoglobulins, and antiviral therapy (Supplementary Appendix 1-Table 8).
B. *The effect of protein supplementation according to nutritional risk:* Malnutrition in critically ill patients is highly prevalent and associated with adverse clinical outcomes. Therefore, nutritional risk assessment is considered important to recognize high nutrition risk earlier and provide targeted nutritional therapy [36]. However, there is a lack of consensus regarding the definition of nutritional risk. The Nutrition Risk in Critically Ill (NUTRIC) score is the first nutritional risk assessment tool developed and validated specifically for ICU patients [37, 38]. The score includes age, APACHE II score, SOFA score, number of comorbidities, days from hospital admission to ICU admission, and IL-6. A modified version of the NUTRIC score, which excludes IL-6, has been validated in observational studies; the total score ranges from 0 to 9, with increasing scores indicating higher nutritional risk [39]. Based on this baseline score, we will conduct a pre-defined subgroup analysis on high versus low NUTRIC patients. Other nutritional risk indicators that will be used are prealbumin (prealbumin ≤ 0.10 g/L considered as an indicator of severe nutritional risk, 0.11–0.15 g/L as mild to moderate risk, and > 0.15 g/L as no risk) [40, 41], serum albumin (35 g/L considered as a cutoff value), baseline urine urea nitrogen (using the median of the cohort as a cutoff value), baseline nitrogen balance (positive versus negative balance) [42], and SARC-F (1–3 versus ≥ 4). Some of these tests are not performed routinely, but we will not impute for missing nutritional risk indicators in this analysis.
C. *The effect of protein supplementation across different BMI strata:* With obesity increasing worldwide, there is also a rise in the prevalence of obesity in patients admitted to the ICU. Despite being associated with comorbid conditions, obesity has no independent effect on the outcome of critical illness other than increased ICU length of stay and increased severity of illness [43]. On the other hand, underweight patients may have a higher risk of mortality, possibly due to inadequate nutritional reserves to compensate for the stress of critical illness [44]. Studies on the optimal dose and timing of enteral protein in critically ill patients according to their BMI are scarce [45]. We will perform subgroup analyses stratified by BMI categories and evaluate the effect of protein intake on their outcomes. By definitions of the National Institutes of Health and World Health Organization, a person with a BMI < 18.5 kg/m^2^ is underweight, 18.5 to 25 kg/m^2^ has normal weight, 25 to 29.9 kg/m^2^ is overweight, 30 to 39.9 kg/m^2^ is obese, and ≥ 40 kg/m^2^ is morbidly obese [46].
D. *Other analyses.* We will perform another analysis on patients who received corticosteroids (at any dose) during ICU stay and stratify them between high and low hydrocortisone-equivalent doses using a median cutoff of 300 mg to examine whether protein supplementation improves the outcome in these patients.

Additional details about the SAP are available in Supplementary Appendix 2.

#### Trial status

The first patient was enrolled in September 2020. As of January 2024, 1578 patients have been enrolled from 17 centers in Saudi Arabia and Kuwait. The first interim analysis was conducted on November 28, 2022, after a 1/3 accrual period when 833 patients had completed their 90-day outcome. In addition, an unplanned interim analysis was carried out on June 7, 2023, in response to the publication of the EFFORT-protein trial (high versus moderate protein), which found no difference in the primary outcome but a worse outcome in subgroups of patients with AKI and high SOFA [47]. On the advice of the Steering Committee and the DSMB, enrollment of patients with AKI (as defined in the EFFORT trial) was paused on February 2, 2023, with the recommendation to conduct an unplanned interim analysis that included subgroups by AKI and SOFA. After reviewing the data from the unplanned interim analysis, the DSMB recommended that the trial be continued with the same inclusion and exclusion criteria and procedures indicated in the protocol before restricting enrolment.

## Discussion

The REPLENISH trial examines the effectiveness of supplemental protein (addition of supplemental enteral protein at 1.2 g/kg/day to the standard amount of protein (maximum 1.2 g/kg/day) from the primary formula) versus the standard amount of protein (maximum 1.2 g/kg/day) starting day 5 of ICU admission. It addresses a major evidence gap in critical care nutrition and will contribute to future clinical practice guidelines.

The SAP of REPLENISH trial described here specifies the statistical methods for evaluating primary and secondary outcomes and pre-defines the covariates for adjusted analyses and the procedures for dealing with missing data. Applying this SAP will facilitate unbiased analyses of clinical data and increase the robustness of its results and conclusions.

### Supplementary Information

**Supplementary Material 1.**

**Supplementary Material 2.**

## Data Availability

The data that will support the findings of this study are available from the corresponding author upon reasonable request as per the regulations of King Abdullah International Medical Research Center (KAIMRC).

## Abbreviations

RCT: Randomized clinical trial
CONSORT: Consolidated Standards of Reporting Trials
CI: Confidence interval
ICU: Intensive care unit
LOS: Length of stay
RR: Relative risk
SAP: Statistical analysis plan
