# Supplementary material for: Statistical analysis plan for the replacing protein via enteral nutrition in a stepwise approach in critically ill patients (REPLENISH) randomized clinical trial
Source: Trials. 2024 May 2;25:296. doi: 10.1186/s13063-024-08105-w (PMC11064302; doi:10.1186/s13063-024-08105-w)
Supplement: Supplementary file 1 — Supplementary Material 1. [file 13063_2024_8105_MOESM1_ESM.docx]

**Supplementary appendix to Replacing Protein via Enteral Nutrition in a Stepwise Approach in Critically Ill Patients (REPLENISH): Statistical Analysis Plan for the Randomized Controlled Trial**

**Contents**

**Supplemental Table 1.** Baseline characteristics

**Supplemental Table 2.** Summary of interventions and co-interventions.

**Supplemental Table 3.** Primary outcome.

**Supplemental Table 4.** Secondary and safety outcomes.

**Supplemental Table 5.** Components of the SARC-F and EQ-5D-5L in the supplemental protein group and the control group

**Supplemental Table 6.** Summary of Protocol Violations and Serious Adverse Events.

**Supplemental Table 7.** Subgroup analyses.

**Supplemental Table 8.** Additional information for COVID-19 patients.

**Supplemental Figure 1.** Kaplan Meier Survival Curve for overall survival

**Supplemental Figure 2.** Serial parameters among patients in the supplemental protein group and control group.

**Supplemental Table 1:** Baseline characteristics.

| **Variable** | **Supplemental protein group**  **N =** | **Control group**  **N =** |
| --- | --- | --- |
| **Age** (yrs), mean ± SD |  |  |
|  |  |  |
| **Female sex**, n (%) |  |  |
|  |  |  |
| **Height** (cm), mean ± SD |  |  |
|  |  |  |
| **Weight** (kg), mean ± SD |  |  |
|  |  |  |
| **BMI** (kg/m^2^), mean ± SD |  |  |
|  |  |  |
| **Diabetes**, n (%) |  |  |
|  |  |  |
| **Sepsis**, n (%) |  |  |
|  |  |  |
| **Traumatic brain injury**, n (%) |  |  |
|  |  |  |
| **Admission category**, n (%) |  |  |
| Post-operative non-trauma |  |  |
| Medical |  |  |
| Trauma |  |  |
|  |  |  |
| **Suspected or confirmed COVID-19**, n (%) |  |  |
|  |  |  |
| **Chronic comorbidities**, n (%) |  |  |
| Chronic pulmonary disease |  |  |
| Chronic liver disease |  |  |
| Chronic renal disease |  |  |
| Chronic cardiovascular disease |  |  |
| Immunosuppression |  |  |
|  |  |  |
| **Mechanical ventilation**, n (%) |  |  |
|  |  |  |
| **Vasopressor therapy**, n (%) |  |  |
|  |  |  |
| **Renal replacement therapy**, n (%) |  |  |
|  |  |  |
| **Systemic corticosteroid use**, n (%) |  |  |
|  |  |  |
| **Neuromuscular blockade use**, n (%) |  |  |
|  |  |  |
| **Sedative use**, n (%) |  |  |
|  |  |  |
| **APACHE II**, mean ± SD |  |  |
|  |  |  |
| **Mean arterial pressure** (mmHg), mean ± SD |  |  |
|  |  |  |
| **GCS**, mean ± SD |  |  |
|  |  |  |
| **PaO2/FiO2 ratio**, mean ± SD |  |  |
|  |  |  |
| **SOFA Score Day 1**, mean ± SD |  |  |
|  |  |  |
| **Inclusion blood glucose** ( mmol/L), mean ± SD |  |  |
|  |  |  |
| **Bilirubin** (μmol/L)**,** mean ± SD |  |  |
|  |  |  |
| **Creatinine** (µmol/L), mean ± SD |  |  |
|  |  |  |
| **Blood urea nitrogen** (mmol/L), mean ± SD |  |  |
|  |  |  |
| **Platelets** (10^9^/L), mean ± SD |  |  |
|  |  |  |
| **INR**, mean ± SD |  |  |
|  |  |  |
| **Albumin** (g/L), mean ± SD |  |  |
|  |  |  |
| **Pre-albumin** (g/L), mean ± SD |  |  |
|  |  |  |
| **In Covid positive patients** |  |  |
| Procalcitonin |  |  |
| Ferritin |  |  |
| Interleukin-6 |  |  |
| Lactate |  |  |
|  |  |  |
| **Highest level of mobility,** n (%) |  |  |
| Bedbound |  |  |
| Edge of bed |  |  |
| Chair |  |  |
| Walking |  |  |
|  |  |  |
| **SARC-F** |  |  |
| Difficulty in lifting and carrying 10 pounds (4.5 kgs), n (%) |  |  |
| Difficulty in walking across a room, n (%) |  |  |
| Difficulty in transferring from a chair or bed, n (%) |  |  |
| Difficulty in climbing a flight of 10 stairs, n (%) |  |  |
| Number of falls in the past year, n (%) |  |  |

BMI: body mass index; COVID: Coronavirus disease; APACHE: Acute Physiology and Chronic Health Evaluation, INR: international normalized ratio, GCS: Glasgow coma scale, PaO2/FiO2 ratio: the ratio of the partial pressure of oxygen to the fraction of inspired oxygen, SOFA: Sequential Organ Failure Assessment, SARC-F: Strength, assistance with walking, rising from a chair, climbing stairs, and falls

For all percentages, the denominator is the total number of subjects in the group. Chi-square test will be used to calculate the P-value.

**Supplemental Table 2:** Summary of interventions and co-interventions.

| **Variable** | **Supplemental protein group**  **N =** | **Control group**  **N =** | **P-value** |
| --- | --- | --- | --- |
| **Calculated daily energy requirement** (kcal), mean ± SD |  |  |  |
|  |  |  |  |
| **Pre-randomization (Day 1-4)** |  |  |  |
| Daily energy intake (Calories),mean± SD |  |  |  |
| Enteral nutrition (Kcal), mean ± SD |  |  |  |
| Propofol (Kcal), mean ± SD |  |  |  |
| Intravenous dextrose (Kcal), mean ± SD |  |  |  |
| Citrate (Kcal), mean ± SD |  |  |  |
| Energy from parenteral nutrition (Kcal), mean ± SD |  |  |  |
| Daily energy intake (Calories/kg), mean ± SD |  |  |  |
|  |  |  |  |
| **Post-randomization (The intervention period starting day 5)** |  |  |  |
| Daily energy intake (without energy from Supplemental protein) (Calories), mean ± SD |  |  |  |
| Daily energy intake (with energy from Supplemental protein) (Calories), mean ± SD |  |  |  |
| Enteral nutrition (Kcal), mean ± SD |  |  |  |
| Propofol (Kcal), mean ± SD |  |  |  |
| Intravenous dextrose (Kcal), mean ± SD |  |  |  |
| Citrate (Kcal), mean ± SD |  |  |  |
| Energy from parenteral nutrition (Kcal), mean ± SD |  |  |  |
| Energy from Supplemental protein (Kcal), mean ± SD |  |  |  |
| Daily energy intake (Calories/kg), mean± SD |  |  |  |
|  |  |  |  |
| **Calculated daily protein requirement** (gram), mean ± SD |  |  |  |
|  |  |  |  |
| **Pre-randomization (Day 1-4)** |  |  |  |
| Daily protein intake (gram), mean± SD |  |  |  |
| Enteral protein from the enteral formula (Kcal), mean ± SD |  |  |  |
| Enteral supplemental protein (gram), mean ± SD |  |  |  |
| Protein from parenteral nutrition (Kcal), mean ± SD |  |  |  |
| Daily protein intake (gram/kg), mean± SD |  |  |  |
|  |  |  |  |
| **Post-randomization (The intervention period starting day 5)** |  |  |  |
| Daily protein intake (gram), mean ± SD |  |  |  |
| Enteral protein from the enteral formula (Kcal), mean ± SD |  |  |  |
| Enteral supplemental protein (gram), mean ± SD |  |  |  |
| Protein from parenteral nutrition (Kcal), mean ± SD |  |  |  |
| Daily protein intake (gram/kg), mean ± SD |  |  |  |
|  |  |  |  |
| **Type of enteral formulas on day 1,** n (%) |  |  |  |
| With a specific disease indication |  |  |  |
| Without a specific disease indication |  |  |  |
|  |  |  |  |
| **Duration of intervention,** mean ± SD |  |  |  |
|  |  |  |  |
| **Cointerventions received during study period** |  |  |  |
| **Insulin**, n (%) |  |  |  |
| **Use**, n (%) |  |  |  |
| **Dose**, mean ± SD |  |  |  |
| **Prokinetics**, n (%) |  |  |  |
| **Corticosteroid use**, n (%) |  |  |  |
| Maximum daily dose, mean ± SD |  |  |  |
| **Statins**, n (%) |  |  |  |
|  |  |  |  |
| **Blood glucose** (mmol/liter), mean ± SD |  |  |  |
|  |  |  |  |

Disease-nonspecific formulae: Osmolite, Jevity, Promote, Ensure plus, Resourse, Ensure, Resource plus, and Jevity (1.2); Jevity (1.06), Jevity (1.0), Fresubian 2 Cal

Disease-specific formulae: Glucerna, Nutric hepatic, Nepro, Pulmocare, Novasource Renal, Peptamen (1.0), Peptamen (1.2), Suplena, and Oxepa.

All continuous variables will be reported as mean and SD (standard deviation) or median (quartile Q1, Q3) based on normality testing.

**Supplemental Table 3:** Primary outcome.

| **Outcomes** | **Supplemental protein group**  **N =** | **Control group**  **N =** | **P-value** |
| --- | --- | --- | --- |
| Primary analysis of 90-day mortality, n (%) |  |  |  |
| Risk difference (95% CI) |  | |  |
| Relative risk (95% CI) |  | |  |
| Analysis using the Chi-square or Fisher's Exact test-Relative risk (95% CI) |  | |  |
| Generalized mixed effects model with the site as a random effect and with imputation- Relative risk (95% CI) |  | |  |
| Cox proportional analysis- Hazard ratio (95% CI) |  | |  |
| Adjusted analysis Cox proportional analysis*- Hazard ratio (95% CI) |  | |  |

*Adjusted Cox proportional hazard model with site as random effect adjusting for the following a priori-defined factors: APACHE II score, type of admission (medical versus non-medical), presence of acute kidney injury at baseline and COVID-19 status**Supplemental Table 4:** Secondary and safety outcomes.

| **Outcomes** | **Supplemental protein group**  **N =** | **Control group**  **N =** | **Difference (median difference or risk difference), 95% CI** | **Effect (beta estimate or relative risk), 95% CI** | **P-value** | **FDR** |
| --- | --- | --- | --- | --- | --- | --- |
| Days alive at day 90 without life support, mean ± SD |  |  |  |  |  |  |
| Vasopressor free-days, mean ± SD |  |  |  |  |  |  |
| Renal replacement-free days, mean ± SD |  |  |  |  |  |  |
| Ventilation-free days, mean ± SD |  |  |  |  |  |  |
| Days alive and out of the hospital at day 90, mean ± SD |  |  |  |  |  |  |
| Bacteremia, n (%) |  |  |  |  |  |  |
| New or progression of skin sacral pressure ulcers, n (%) |  |  |  |  |  |  |
| Stage I: Non-blanchable erythema, n (%) |  |  |  |  |  |  |
| Stage II: Partial thickness, n (%) |  |  |  |  |  |  |
| Stage III: Full thickness skin loss, n (%) |  |  |  |  |  |  |
| Stage IV: Full thickness tissue loss, n (%) |  |  |  |  |  |  |
| SARC-F score at day 90, mean ± SD |  |  |  |  |  |  |
| EQ-5D-5L index value at day 90, mean ± SD |  |  |  |  |  |  |
| EQ-VAS at day 90, mean ± SD |  |  |  |  |  |  |
|  |  |  |  |  |  |  |
| **Major safety outcomes** |  |  |  |  |  |  |
| New episode of stage 2 or higher acute kidney injury by KDIGO criteria, n (%) |  |  |  |  |  |  |
| Newly confirmed pneumonia, n (%) |  |  |  |  |  |  |
| Grade IV acute gastrointestinal injury, n (%) |  |  |  |  |  |  |
| Bowel ischemia |  |  |  |  |  |  |
| Clinically important gastrointestinal bleeding |  |  |  |  |  |  |
| Ogilvie’s syndrome |  |  |  |  |  |  |
| Abdominal compartment syndrome |  |  |  |  |  |  |
| **Minor safety outcomes**, n (%) |  |  |  |  |  |  |
| Feeding intolerance |  |  |  |  |  |  |
| Diarrhea |  |  |  |  |  |  |
| Refeeding syndrome |  |  |  |  |  |  |

**SARC-F:** Strength, assistance with walking, rising from a chair, climbing stairs, and falls; **EQ-5D-5L:** EuroQoL- 5 Dimension-5 Levels; **EQ-VAS:** EuroQoL visual analogue scale; **KDIGO:** Kidney Disease Improving Global Outcomes, **FDR:** False recovery rate.

All continuous variables will be reported as mean and SD (standard deviation) or median (quartile Q1, Q3) based on normality testing.

**Supplemental Table 5:** Components of the SARC-F and EQ-5D-5L in the supplemental protein group and the control group at day 90.

|  | **Supplemental protein group**  **N =** | **Control group**  **N =** | **P-value** |
| --- | --- | --- | --- |
| **SARC-F screen,** n (%) |  |  |  |
| Difficulty in lifting and carrying 10 pounds (4.5 kgs)? |  |  |  |
| Difficulty in walking across a room? |  |  |  |
| Difficulty in transferring from a chair or bed? |  |  |  |
| Difficulty in climbing a flight of 10 stairs? |  |  |  |
| Number of falls in the past year? |  |  |  |
|  |  |  |  |
| **EQ-5D-5L,** n (%) |  |  |  |
| **Mobility** | | |  |
| Slight problems |  |  |  |
| Moderate problems |  |  |  |
| Severe problems |  |  |  |
| Unable to |  |  |  |
| **Self-care** | | |  |
| Slight problems |  |  |  |
| Moderate problems |  |  |  |
| Severe problems |  |  |  |
| Unable to |  |  |  |
| **Usual activities** | | |  |
| Slight problems |  |  |  |
| Moderate problems |  |  |  |
| Severe problems |  |  |  |
| Unable to |  |  |  |
| **Pain/discomfort** | | |  |
| Slight |  |  |  |
| Moderate |  |  |  |
| Severe |  |  |  |
| Extreme |  |  |  |
| **Anxiety/depression** | | |  |
| Slight |  |  |  |
| Moderate |  |  |  |
| Severe |  |  |  |
| Extreme |  |  |  |
|  |  |  |  |
| **The value for health state (VAS),** mean ± SD |  |  |  |

**SARC-F:** Strength, assistance with walking, rising from a chair, climbing stairs, and falls; **EQ-5D-5L:** EuroQoL- 5 Dimension-5 Levels; **VAS:** visual analogue scale

**Supplemental Table 6:** Summary of Protocol Violations and Serious Adverse Events.

|  | **Supplemental protein group**  **N =** | **Control group**  **N =** |
| --- | --- | --- |
| **Protocol violations, n (%)** |  |  |
| Patient was randomized to one group but feeding started or continued according to the other group, n (%) |  |  |
| Patient enrolled more than 96 hours after ICU admission, n (%) |  |  |
| Patient was randomized but feeding was not started, n (%) |  |  |
| Other, n (%) |  |  |
|  |  |  |
| **Serious Adverse Events, n (%)** |  |  |

**Supplemental Table 7:** Prespecified subgroups. All subgroups are defined based on baseline characteristics. Heterogeneity of the intervention effects on the primary outcome will be evaluated by the test of interaction using log binomial regression. Analyses of prespecified subgroups will be adjusted for multiple testing using the False Discovery Rate (FDR).

| **Subgroup** | **Supplemental protein group**  **N =** | **Control group**  **N =** | **RR (95%CI)** | **P-value** | **P-value for interaction** | **FDR** | **Anticipated effect** |
| --- | --- | --- | --- | --- | --- | --- | --- |
| Admission category**,** n (%) |  |  |  |  |  |  | More effect in medical patients, because of malnutrition |
| Medical |  |  |  |  |  |  |  |
| Surgical |  |  |  |  |  |  |  |
| Trauma |  |  |  |  |  |  |  |
|  |  |  |  |  |  |  |  |
| Sepsis**,** n (%) |  |  |  |  |  |  | More effect in sepsis |
| Yes |  |  |  |  |  |  |  |
| No |  |  |  |  |  |  |  |
|  |  |  |  |  |  |  |  |
| Suspected or confirmed COVID-19**,** n (%) |  |  |  |  |  |  | No difference |
| Yes |  |  |  |  |  |  |  |
| No |  |  |  |  |  |  |  |
|  |  |  |  |  |  |  |  |
| Vasopressor use**,** n (%) |  |  |  |  |  |  | More effect in patients on vasopressors |
| Yes |  |  |  |  |  |  |  |
| No |  |  |  |  |  |  |  |
|  |  |  |  |  |  |  |  |
| Acute kidney injury**,** n (%) |  |  |  |  |  |  | More effect with normal kidney function |
| No AKI |  |  |  |  |  |  |  |
| AKI stage I |  |  |  |  |  |  |  |
| AKI stage II |  |  |  |  |  |  |  |
| AKI stage III |  |  |  |  |  |  |  |
|  |  |  |  |  |  |  |  |
| Liver injury |  |  |  |  |  |  | More effect with normal liver function |
| Yes |  |  |  |  |  |  |  |
| No |  |  |  |  |  |  |  |
|  |  |  |  |  |  |  |  |
| BMI ≤30**,** n (%) |  |  |  |  |  |  | More effect in obese patients |
| BMI >30**,** n (%) |  |  |  |  |  |  |  |
|  |  |  |  |  |  |  |  |
| NUTRIC score 5-9**,** n (%) |  |  |  |  |  |  | More effect with malnutrition |
| NUTRIC score <5**,** n (%) |  |  |  |  |  |  |  |
|  |  |  |  |  |  |  |  |
| SARC-F<4**,** n (%) |  |  |  |  |  |  | More effect with sarcopenia |
| SARC-F ≥ 4**,** n (%) |  |  |  |  |  |  |  |
|  |  |  |  |  |  |  |  |
| SOFA <median, n (%) |  |  |  |  |  |  | More effect in more severely ill patients |
| SOFA ≥median, n (%) |  |  |  |  |  |  |  |

**COVID-19**: Coronavirus disease; **AKI**: Acute kidney injury; **BMI**: Body mass index; **NUTRIC**: NUTrition Risk in the Critically Ill; **SARC-F**: Strength, assistance with walking, rising from a chair, climbing stairs, and falls; **SOFA:** Sequential Organ Failure Assessment

**Supplemental Table 8.** Additional information for COVID-19 patients.

| **Variable** | **Supplemental protein group**  **N =** | **Control group**  **N =** | **P-value** |
| --- | --- | --- | --- |
| **Baseline laboratory tests**, mean ± SD |  |  |  |
| Procalcitonin |  |  |  |
| Ferritin |  |  |  |
| Interleukin-6 |  |  |  |
| Lactate |  |  |  |
| **Medications,** n (%) |  |  |  |
| Prone positioning |  |  |  |
| ECMO |  |  |  |
| Inhaled nitric oxide |  |  |  |
| Tracheostomy |  |  |  |
| Tocilizumab |  |  |  |
| Lopinavir/Ritonavir |  |  |  |
| Interferon alpha |  |  |  |
| Interferon beta |  |  |  |
| Chloroquine/Hydroxychloroquine |  |  |  |

**ECMO:** Extracorporeal membrane oxygenation.

**Supplemental Figure 1.** Kaplan Meier Survival Curve for overall survival

**Supplemental Figure 2.** Serial parameters among patients in the supplemental protein group and control group.

1. Serial measurements of energy and protein
2. Serial measurements of glucose, insulin, nitrogen balance, prealbumin, ammonia, BUN, creatinine and 24-hour urine for urinary urea nitrogen.
