# Supplementary material for: Statistical analysis plan for the replacing protein via enteral nutrition in a stepwise approach in critically ill patients (REPLENISH) randomized clinical trial
Source: Trials. 2024 May 2;25:296. doi: 10.1186/s13063-024-08105-w (PMC11064302; doi:10.1186/s13063-024-08105-w)
Supplement: Supplementary file 2 — Supplementary Material 2. [file 13063_2024_8105_MOESM2_ESM.docx]

**Statistical Analysis Plan for the Replacing Protein via Enteral Nutrition in a Stepwise Approach in Critically Ill Patients (REPLENISH): A Randomized Clinical Trial**

Protocol Version 4.1: June 15, 2022

Clinicaltrials.gov identifier: NCT04475666

Version 1

December 1, 2022

Principal Investigator: _____________________________

Dr. Yaseen Arabi


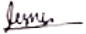


Statistician: _____________________________

Jesna Jose

**Table of Contents**

1. Introduction 4

2. Study outcomes 4

2.1 Primary Outcome 4

2.2 Secondary Outcomes 4

3. Study description 6

3.1 Study Design 6

3.2 Randomization Procedure 7

3.3 Sample Size 7

3.4 Study Interventions 8

3.5 Study Duration 8

3.6 Schedule of Assessments 9

4. Inclusion, exclusion, and withdrawal criteria 9

4.1 Inclusion criteria 9

4.2 Exclusion criteria 10

4.3 Eligible non-randomized 10

5. Study outcomes and definitions 11

6. Study populations 12

7. Statistical considerations 13

7.1 Statistical significance 13

7.2 Handling dropouts and missing data 14

7.3 Adjustment for multiplicity 14

7.4 Statistical software 14

8. Description of tables and figures 15

8.1 Subject disposition 15

8.1.1 Subject disposition 15

8.1.2 Protocol violations / deviations 15

8.2 Baseline and demographic characteristics 15

8.3 Efficacy analysis 16

8.3.1 Analysis of the primary outcome and continuous planning of the trial 16

8.3.2 Secondary analyses of the primary outcome, secondary outcomes and

subgroups. 16

8.4 Safety Analysis 18

8.4.1 Adverse Events (AE) 18

8.4.2 Laboratory parameters 18

8.4.3 Treatment exposure and compliance 18

8.4.4 Prior and concomitant medications and interventions 18

9. Interim report details 19

10. Data monitoring committee charter 20

11. Report generation 23

12. References 24

**LIST OF ABBREVIATIONS**

**APACHE II:**Acute Physiology and Chronic Health Evaluation II

**CI:** Confidence Interval

**CONSORT**: Consolidated Standards of Reporting Trials

**DSMB**: Data and Safety Monitoring Board

**HR:** Hazard Ratio

**ICU:** Intensive Care Unit

**IQR:** Interquartile Range

**LOS:** Length of stay

**RCT:** Randomized Controlled Trial

**RR:** Relative Risk

**RRR:** Relative Risk Reduction

**SAP:**Statistical Analysis Plan

**SOFA:** Sequential-Organ-Failure Assessment

**Introduction**

The purpose of the Statistical Analysis Plan (SAP) is to provide an outline that addresses the protocol objectives with minimized bias or analytical deficiencies.  Specifically, this plan has the following purpose: to prospectively (a priori) outline the types of analyses and data presentations that will addresses the study objectives outlined in the protocol, and to explain in detail how the data will be handled and analyzed, adhering to commonly accepted standards and practices.

The final study report will follow the Consolidated Standards of Reporting Trials (CONSORT) 2010 guidelines for reporting RCTs. [1, 2]

The trial is being conducted according to the standard requirements of Good Clinical Practice E6.[3] This SAP complies with the International Conference on Harmonisation of Technical Requirements for Registration of Pharmaceuticals for Human Use, the "Statistical principles for clinical trials E9" report and "Structure and content of clinical study report E3".[4, 5] This statistical analysis plan identifies the procedures to be applied to the primary and secondary analyses once trial data validation is complete. All analyses were prospectively defined, and the SAP will be finalized before the final analysis. The SAP was written by the Principal Investigator and members of the Steering committee. Participant recruitment is expected to be completed in December 2024.

**Study outcomes**

- Primary outcome

90-day all-cause mortality defined as death by day 90 from the ICU admission date.

- Secondary outcomes
- Days alive at day 90 without life support, which will be calculated if a patient is alive and free of vasopressor use, invasive mechanical ventilation and renal replacement therapy until day 90. We will report as a supplementary analysis the components of this composite outcome: vasopressor-free days, invasive mechanical ventilation-free days, and renal replacement-free days. Patients who die during the 90-day follow-up will be assigned zero free days.
- Days alive and out of the hospital at day 90 will be calculated by using the days from alive hospital discharge to day 90. Patients who die during 90-day follow-up will be assigned zero free days.
- Bacteremia defined as positive blood cultures, excluding those which are considered contaminant organisms, until two days post ICU discharge.
- New or progression of skin sacral pressure ulcers in ICU, using the definitions of the National Pressure Ulcer Advisory Panel which include stage I: non-blanchable erythema, stage II: partial-thickness skin loss, stage III: full-thickness skin loss, and stage IV: full-thickness tissue loss.
- Functional assessment using SARC-F (Strength, assistance with walking, rising from a chair, climbing stairs, and falls) score for sarcopenia at day 90.
- EuroQoL 5-Dimension 5-Level (EQ-5D-5L) index value and EQ visual analog scale (EQ-VAS) at day 90. The EQ-5D-5L has five dimensions (mobility, self-care, usual activities, pain or discomfort, and anxiety/depression) with five levels of severity (no problems, slight problems, moderate problems, severe problems, extreme problems). A higher score indicates a worse condition. The scores of each patient will be first converted into a single index value. The EQ-5D-5L index value will be calculated using the Kingdom of Saudi Arabia value sets, if they become available at the time of analysis, otherwise we will use the United States EQ-5D-5L value sets. Patients who have died at 90-days follow-up will be assigned zero values in both index value and EQ-VAS. Data will also be presented for survivors only.
- *Safety outcomes* are classified into major and minor safety outcomes.

Major safety outcomes include

- New episode of stage 2 or higher of acute kidney injury by KDIGO (Kidney Disease Improving Global Outcomes) criteria after enrollment. This is defined as new initiation of renal replacement therapy after randomization, an increase in creatinine by ≥2.0 folds compared to the baseline creatinine (the lowest available value before randomization) or urine output <0.5 ml/kg/hr on any given day post-randomization.
- Newly confirmed pneumonia according to the modified CDC criteria.
- Grade IV acute gastrointestinal injury, including any bowel ischemia with necrosis, clinically important gastrointestinal bleeding, Ogilvie's syndrome, and abdominal compartment syndrome.

Minor safety outcomes include

- Feeding intolerance: defined as vomiting or large gastric residual volume (GRV) (GRV ≥500 ml/24 h) on a single calendar day.
- Diarrhea defined as having three or more loose or liquid stools per day with a stool weight >200–250 g/day (or > 250 ml/day).
- Refeeding syndrome defined as a fall in serum phosphate below 0.65 mmol/L within 72 hours of starting nutritional support and the drop being >0.16 mmol/L from a previously recorded reading during ICU stay.

**Study description**

- Study Design

REPLENISH trial is a multicenter, parallel, randomized controlled, open label trial.

The REPLENISH (Replacing Protein Via Enteral Nutrition in a Stepwise Approach in Critically Ill Patients) trial is an open-label, multicenter RCT that is conducted in ICUs in Saudi Arabia and Kuwait (Supplementary file Table 1). The study has been approved by the Institutional Review Boards of all the participating sites and sponsored by King Abdullah International Medical Research Center, Riyadh Saudi Arabia (RC19/414/R). It has been registered at ClinicalTrials.gov (NCT04475666).

Medical, surgical and trauma patients will be screened on day 4 of ICU stay up to the morning of day 5. Adult mechanically ventilated patients (≥18 years old) on enteral nutrition who are unlikely to be discharged from the ICU on the next day will be included in the study. Eligible patients will be randomized to the supplemental protein group (range 2-2.4 g/kg/day) or the control group (0.8-1.2 g/kg/day).

The study intervention starts from ICU day 5 (12 midnight) and is continued till meeting any of the following criteria: death, ICU discharge or day 90 in ICU, premature stopping of feeding due to brain death or palliative care plan, or initiation and tolerance of full oral feeding for more than 24 hours, whichever comes first. Patients are followed up daily until day 90 if in the ICU or until ICU discharge and then at day 90.

- Randomization Procedure

Enrolled patients will be randomized through a web-based system at a 1:1 ratio to Supplemental protein group or Control group using permuted variable undisclosed block sizes. Randomization will be stratified by the trial site, the use of renal replacement therapy at the time of randomization and whether the patient is a suspected or confirmed case of COVID-19.

- Sample Size

The study sample size of 2502 patients will have 80% power to detect a 5% absolute risk reduction in 90-day mortality from 30% to 25%.

We anticipate a baseline 90-day mortality of 30% and an absolute risk reduction of 5% with the high-protein intervention. The baseline risk was estimated based on a similar cohort from the Permissive Underfeeding or Standard Enteral Feeding in Critically Ill Adults (PermiT trial) and Pantoprazole in patients at risk for gastrointestinal bleeding in the ICU (SUP-ICU) trials. In the PermiT trial which included patients from 7 sites in Saudi Arabia and Canada,[6] 715 patients received mechanical ventilation for >4 days, and 209 died by day 90 (29.3%). In the SUP-ICU trial,[7] 48% (1571/3282) of all included patients were mechanically ventilated on day 4. Of these, 34% (530/1571) had died on day 90. The SUP-ICU trial enrolled acutely admitted ICU patients with at least one risk factor for GI bleeding in 33 ICUs in 5 countries in Northern Europe. The treatment effect in REPLENISH (5% absolute risk reduction) was based on a propensity-score adjusted analysis which showed an odds ratio for the association of high protein compared to a moderate protein of 0.80 (95% CI 0.56, 1.16, p=0.24).[8] The final analysis of the primary outcome will be based on two-sided alpha (α) of 0.05 and power (1- β) = 0.80. Based on these assumptions, we need 1251 patients in each group (2502 in both groups).

- Study Interventions

Supplemental enteral protein (1.2 g/kg/day) added to standard enteral nutrition to achieve a high amount of enteral protein (range of 2-2.4 g/kg/day) or no supplemental enteral protein to achieve a moderate amount of enteral protein (0.8-1.2 g/kg/day).

- Study Duration

The study intervention will continue until meeting any of the following criteria: death, ICU discharge or day 90 in ICU, premature stopping of feeding due to brain death or palliative care plan whichever comes first, initiation and tolerating of full oral feeding for more than 24 hours (i.e., treating physicians feel that enteral nutrition is no longer required). In these situations, the study intervention will no longer be followed, and nutrition will be at the discretion of the treating teams but outcome data will be collected.

- Schedule of Assessments

*Signifies days 15, 16,17,18,19,20,22,23,24,25,26,27 and 29 to 89; SARC-F: Screening test for sarcopenia; EQ-5D-5L: EuroQol

**Inclusion, exclusion, and withdrawal criteria**

**Study population**

All the patients will be screened for the eligibility criteria (Table 1) on ICU calendar day 4, up to the morning of ICU calendar day 5. The ICU admission calendar day is considered ICU day 1.

- **Inclusion criteria**

1. Age ≥18-years old.
2. The patient is started on enteral nutrition via feeding tube (naso/oro-gastric, naso/oro-enteral, gastrostomy or jejunostomy tubes).
3. The patient is on invasive mechanical ventilation and unlikely to be discharged from ICU next day.

- **Exclusion criteria**

1. Lack of commitment to full life support or brain death. Patients with “Do-Not-Resuscitate” order but with commitment to ongoing life support can be enrolled.
2. The patient is on any amount of parenteral nutrition (PN) in ICU at the time of screening, whether PN is used alone or in combination with enteral nutrition. Non-nutritional calories (dextrose, propofol, citrate) not considered as PN.
3. The patient has received an average protein of more than 0.8 g/kg/day in the first 4 ICU days.
4. The patient is fed entirely through oral route – i.e., those who are eating.
5. The patient has hepatic encephalopathy or Child C liver cirrhosis
6. The patient is admitted because of burn.
7. The patient has an inherited defect of amino acid metabolism.
8. The patient has allergy to protein supplement.
9. Pregnancy.
10. Prisoners or those undergoing forced treatment.

***Loss-to-follow up*** :Patients will be followed post ICU discharge (without further intervention) to document hospital vital status. However, if the randomized patient at any point decides to withdraw from the trial intervention at the request of either the patient himself, family, or the treating physician, the data will be included in the group to which they were allocated as per the intention-to-treat principle and the reason of withdrawal will be documented.

**Study outcomes and definitions**

| **Variables** | **Outcomes** | **Definitions** |
| --- | --- | --- |
| **Primary outcome** | 90-day all-cause mortality | Death within 90 days from ICU admission |
| **Secondary outcomes** | Days alive at day 90 without life support | Without use of vasopressor/inotropic support, invasive mechanical ventilation and/or renal replacement therapy) |
|  | Days alive and out of hospital at day 90 |  |
|  | Bacteremia | Positive blood cultures until 2 days post ICU |
|  | New or progression of skin pressure ulcers in sacral areas | Skin assessment staging  Stage I: Non-blanchable erythema  Stage II: Partial thickness  Stage III: Full thickness skin loss  Stage IV: Full thickness tissue loss. |
|  | Day 90 Functional assessment | SARC-F screen for sarcopenia |
|  | Day 90 functional assessment | Evaluate by EuroQoL (EQ)-5D-5L) |
| **Safety outcomes** | | |
| **Major safety outcomes** | New episode of stage 2 or higher acute kidney injury by KDIGO criteria after enrollment | Stage 2: Increase in creatinine 2.0 to 2.9 multiplied by baseline serum creatinine OR urine output <0.5 ml/kg/hr for ≥ 12 hours  Stage 3: Increase in creatinine ≥ 3.0 multiplied by baseline serum creatinine OR increase to > 4.0 mg/dl (353.6 micromol/L) OR new renal replacement therapy OR urine output <0.3 ml/kg/hr for 24 hours OR anuria for > 24 hours |
|  | Pneumonia defined as episodes of newly confirmed pneumonia according to the modified CDC criteria | Two or more serial chest radiographs with at least one of the following:   - New or progressive and persistent infiltrate - Consolidation - Cavitation   AND  At least one of the following:   - Fever (>38°C) with no other recognized cause - Leukopenia (white cell count < 4 x 10^9^ /l) or leukocytosis (white cell count >12 x 10^9^ /l)   AND  At least two of the following:   - New onset of purulent sputum or change in character of sputum, or increased respiratory secretions or increased suctioning requirements - New onset or worsening cough, or dyspnea, or tachypnea - Rales or bronchial breath sounds - Worsening gas exchange (hypoxemia, increased oxygen requirement, increased ventilator demand) |
|  | Grade IV Acute Gastrointestinal injury (AGI) | **Bowel ischemia** defined as any of the following:   - Absent blood flow in one of the main arteries supplying the bowel with evidence of bowel wall compromise on an imaging study (CT angiography, angiography, or magnetic resonance angiography) - Presence of endoscopy criteria for colonic ischemia according to the Favier classification system (stage I, petechiae; stage II, petechiae and superficial ulcers; and stage III, necrotic ulcers and polypoid lesions) - Evidence of bowel ischemia during surgery. |
|  |  | **Clinically important gastrointestinal bleeding** defined as overt gastrointestinal bleeding and at least one of the following four features within 24 hours of gastrointestinal bleeding (in the absence of other causes) in the intensive care unit   - Spontaneous drop of systolic blood pressure, mean arterial pressure or diastolic blood pressure of 20 mmHg or more - Start of vasopressor or a 20% increase in vasopressor dose - Decrease in hemoglobin of at least 2 g/dl (1.24 mmol/l) or - Transfusion of two units of packed red blood cells or more. |
|  |  | **Ogilvie’s syndrome** defined as bowel dilatation if colonic diameter exceeds 6 cm (greater than 9 cm for cecum) or small bowel diameter exceeds 3 cm, diagnosed either on plain abdominal X-ray or CT scan without underlying mechanical obstruction or other organic cause |
|  |  | **Abdominal compartment syndrome** is defined as a persistent intra-abdominal pressure (IAP) of more than 20 mmHg accompanied by new organ dysfunction or failure |
| **Minor safety outcomes** | Feeding intolerance | Vomiting or large gastric residual volume (GRV) ≥500 ml |
|  | Diarrhea | Three or more loose or liquid stools per day with a stool weight greater than 200–250 g/day (or greater than 250 ml/day) |
|  | Refeeding syndrome | Serum phosphate below 0.65 mmol/L within 72 hours of starting intervention  Or  A drop in serum phosphate by > 0.16 mmol/L from a previously recorded reading within 72 hours of starting intervention |

**Study populations**

**Modified Intention-to-treat population**

The primary analyses will be performed on the modified intention-to-treat population consisting of all enrolled patients whether they received the allocated interventions or not. Post-enrollment exclusion from the modified intention-to-treat population will be limited to the cessation of study procedures due to withdrawal of consent. The data of these patients would only remain in the analyzed dataset if the patient or surrogate decision-maker consented to use trial data. Patients will also be excluded post-enrollment from the modified intention-to-treat population if the eligibility criteria were found not have been met and study interventions were not started. Censoring dates will only occur in case of "real" loss to follow-up (i.e., discharged patients with no information beyond some point in time). In that case, the date of censoring will be the last day of contact or the date of hospital discharge if no other information is available.

| **Endpoints / Analysis** | **Populations / Analysis sets** |
| --- | --- |
| Demographic and Baseline Characteristics | Modified intention-to-treat population |
| Efficacy Analysis | Modified intention-to-treat population |
| Safety Analysis | Modified intention-to-treat population |

**Statistical considerations**

- Statistical significance

Statistical tests will be performed using a two-sided alpha value of 5% to denote significance level.

As appropriate, the Chi-square test or Fisher’s exact test will be used to compare the categorical variables, which will be reported as numbers and percentages. Student’s t-test or the Mann–Whitney U Test will be used as appropriate to compare the continuous variables, which will be reported as means and standard deviations or as medians and interquartile ranges. Shapiro–Wilk test will be used to test whether the data follows normal distribution or not.

| **Variables** | **Test** |
| --- | --- |
| Baseline characteristics | No statistical comparisons |
| Intervention and co-interventions | Chi-square, Fisher’s exact test, Mann Whitney U test, t-test as applicable.  For serial values: generalized linear mixed effect models. |
| Primary outcome | 1. Primary analysis: generalized mixed effects model with adjustment to stratification variables. Report risk difference and relative risk. 2. Secondary analyses: 3. Chi-square or Fisher’s exact test 4. Sensitivity analyses using generalized mixed effects model with adjustment to stratification variables and multiple imputations and worst-case and best-case scenarios 5. Cox proportional analysis 6. Kaplan Meier curves |
| Secondary outcomes | 1. Categorical variables: generalized mixed effects model with adjustment to stratification variables. Report risk difference and relative risk. 2. Continuous variables: linear regression model and van Elteren test as appropriate. The results will be reported as median and mean differences and 95% CI. For HRQoL: sensitivity analysis using multiple imputations 3. Adjust for multiplicity by False Discovery Rate (FDR) |
| Safety Outcomes | Generalized mixed effects model with adjustment to stratification variables. Report risk difference and relative risk. |
| Subgroup analyses | Generalized mixed effects model with adjustment to stratification variables. Report risk difference and relative risk, tests of interaction and FDR |

- Handling dropouts and missing data

To address the missing primary outcomes (loss-to-follow-up), we will perform sensitivity analyses using multiple imputations and worst-case and best-case scenarios.[9]. Otherwise, missing data will not be imputed.

- Adjustment for multiplicity

To adjust for multiple testing , we will use the false discovery rate (FDR) as described by Benjamini and Hochberg method.[10]

- Statistical software

All statistical analyses will be conducted using the SAS software version 9.4 (SAS Institute, Cary, NC, USA).

**Description of tables and figures**

- Subject disposition
- Subject disposition

The number and percentage of patients randomized to each group will be reported. We will report the number of patients who received the allocated intervention, withdrawn/lost-to-follow-up with reasons and included in the final analysis. We will report the number of patients who were screened, met inclusion or exclusion criteria, were enrolled, and were eligible but not enrolled. Reasons for the exclusion of non-included patients will be reported. We will also report the number of patients who were randomized to each group, received the allocated interventions and had primary outcome data. A flow diagram will be constructed according to the CONSORT guidelines.

- Protocol violations / deviations

We will summarize the protocol violations or deviations by treatment group using number (n) and percentage (%).

- Baseline and demographic characteristics

We will summarize and report the demographics and baseline clinical characteristics using descriptive statistics for patients randomized to the Replenish protein group and Standard protein group. Baseline characteristics will be summarized in for the two study groups comprising age, sex, admission category (medical, postoperative (non-trauma) and trauma (postoperative and non-operative)), Acute Physiology and Chronic Health Evaluation (APACHE) II score and chronic health points (defined as per the APACHE II system). Baseline data (pre-randomization ICU day 4) will include morning blood glucose, infusions of sedatives and neuromuscular blockers and systemic corticosteroid use. The sequential Organ Failure Assessment (SOFA) score for each organ system will also be reported on day 4. We will also present the pre-morbid functional assessment using the SARC-F screen for sarcopenia. Categorical variables will be summarized by treatment group using number (n) and percentage (%). Descriptive statistics such as mean, standard deviation (SD) or median (Q1, Q3) will be calculated for continuous variables.

- Efficacy analysis
- Analysis of the primary outcome and continuous planning of the trial

The primary outcome will be compared between the two groups using generalized mixed effects model with adjustment to stratification variables.[11] This approach of adjusting primary analysis for stratification variables has been suggested to avoid an unnecessary loss in power.[11] Results will be reported as risk difference (RD) and relative risk (RR) with 95% confidence intervals (CI). We will perform a secondary analysis using Chi-square or Fisher's Exact test. To address the missing primary outcomes (loss-to-follow-up), we will perform sensitivity analyses using multiple imputations and worst-case and best-case scenarios.[9] We will also use the unadjusted Cox proportional hazard model as a secondary analysis, censoring by the last follow-up date, and the results will be reported as hazard ratio (HR) and 95% CI. The distributions of time to death will be compared using Kaplan-Meier survival curves and a log-rank test. The summary of statistics that will be performed on the primary and secondary outcomes was given in a Table in statistical consideration section.

- Secondary analyses of the primary outcome, secondary outcomes and subgroups.

Secondary categorical outcomes will also be compared in the intention-to-treat cohort using generalized mixed effects model with adjustment to stratification variables. Results will be reported as RD and 95% CI. Continuous data such as EQ-5D-5L index value, EQ-VAS and SARC-F score collected at 90 days post-randomization will also be analyzed using linear regression model and van Elteren test as appropriate with adjustment to stratification variables. The results will be reported as median and mean differences and 95% CI. Because data on EQ-5D-5L index value and EQ-VAS could be missing for some patients, we will conduct sensitivity analysis using multiple imputations.

Outcomes such as Bacteremia and New or progression of skin sacral pressure ulcers will be summarized. Days alive at day 90 without life support, Days alive and out of the hospital at day 90, ICU-free days at day 90, ventilation-free days at day 90, renal replacement therapy-free days at day 90 and vasopressor-free days at day 90 will also be summarized using median (Q1, Q3).

**Subgroup analyses**

Subgroup analysis will be performed for the primary outcome in the subgroups determined at baseline. Results will be reported using RR and 95% CIs, and the multivariable logistic regression will be used to report the results of tests of interactions for these subgroups. We will evaluate the effect of the intervention within the following subpopulations:

- Medical vs. postoperative vs. trauma
- Admission diagnosis of sepsis versus no sepsis
- Vasopressor use at the time of enrollment versus none
- Acute kidney injury at enrollment (4 KDIGO groups: 0, 1, 2, 3)
- COVID-19 versus no COVID-19
- BMI of ≤30 or >30 kg/m^2^
- High nutritional risk defined as a NUTRIC score of 5–9 and low nutritional risk as a NUTRIC score of 0–4
- SARC-F score of <4 or ≥4

**Sensitivity analyses**

we will perform sensitivity analyses for the primary outcome between the Replenish protein group and Standard protein group using multiple imputations and worst-case and best-case scenarios.

Since data on EQ-5D-5L index value and EQ-VAS could be missing for some patients, we will conduct sensitivity analysis using multiple imputations.

- Safety Analysis
- Adverse Events (AE)

Serious adverse events will be summarized by treatment group using number (n) and percentage (%). Relationship with treatment group will also be summarized.

Outcomes such as New episode of stage 2 or higher acute kidney injury by KDIGO criteria, Newly confirmed pneumonia, Grade IV acute gastrointestinal injury, Bowel ischemia, Clinically important gastrointestinal bleeding, Ogilvie’s syndrome, Abdominal compartment syndrome, Feeding intolerance, Diarrhea and Refeeding syndrome will be summarized.

- Laboratory parameters

We will summarize Inclusion blood glucose, Bilirubin, Creatinine, Blood urea nitrogen, Platelets, INR, Albumin and Pre-albumin.

Figures will be presented for the serial measurements of glucose, insulin, nitrogen balance, prealbumin, ammonia, BUN, creatinine, 24-hour urine for urinary urea nitrogen

- Treatment exposure and compliance

Serial measurements of energy and protein exposure will be summarized graphically.

- Prior and concomitant medications and interventions

We will report the estimated and administered energy and protein requirements for each group. Energy intake will include energy from enteral nutrition (including those from protein in the primary formula) in addition to those from intravenous dextrose, citrate, propofol and parenteral nutrition (if any). Energy from supplemental protein will not be counted as part of the total energy. Protein intake includes the protein from the primary formula in both groups, the supplemental protein in the Supplemental protein group and parenteral protein (if any). Energy and protein will be reported as kcal/kg based on actual body weight for patients with a body mass index (BMI) of <30 kg/m2 and adjusted body weight for those with a BMI ≥30 kg/m2. To ensure that energy and protein intake data are collected for complete 24-hour periods, nutrition data will not be included for the last day of intervention if the duration of intervention on that day is less than 24 hours.

We will report daily blood glucose, serum creatinine, and urine output by group. We will compare serial weights and the highest mobility level during the ICUstay. We will also compare serial prealbumin, albumin, ammonia, blood urea nitrogen, 24-hour urine for urinary urea nitrogen, lowest potassium level, lowest magnesium level, lowest phosphate level, aspartate transaminase, alanine aminotransferase and international normalized ratio. We will report the average daily insulin dose during the ICU stay. We will also report the use of corticosteroids and statins in the two groups during the study period.

Categorical variables will compared using the Chi-square test. Continuous variables will be compared using Student's t-test or the Wilcoxon-Mann-Whitney test, as judged appropriate by normality testing. For serial measurements, we will test the change over time and the difference between the two groups over time using generalized linear mixed-effect models. These will be graphically represented.

- **Interim report details**

**DSMB & Interim analyses**

Interim analyses will be conducted when 33% and 67% of the sample size (2502 patients) are achieved. The study has two biostatisticians, one who will be involved in study design and analysis and the other in generating a closed report with unblinded group data. The first interim analysis will be conducted soon when 833 patients have completed their 90-day outcome. The interim test statistics will be conducted for the primary endpoint of 90-day all-cause mortality and safety outcomes. We will consider a p-value of <0.01 for safety and a p-value of <0.001 for effectiveness as early stopping criteria. There will be no plans to terminate the trial for futility. We will use a group sequential α-spending function, calculated using the O'Brien–Fleming method, with two-sided symmetric bounds, and the final p-value will be considered at 0.048.

- **Data monitoring committee charter**

The Data Safety Monitoring Board, which is responsible for reviewing reports regarding the safety of the study patients and protocol adherence, may make recommendations to continue or terminate the study on the basis of the results from the interim analysis. The Study Steering Committee will meet frequently at the initial stages of the study and on a bimonthly basis thereafter to ensure the correct implementation of the protocols.

**Sub-Studies**

1. ***REPLENISH-COVID sub-study:*** We will evaluate the effect of high versus moderate protein on the subgroup of suspected or confirmed COVID-19 patients at the time of enrollment. Critically ill patients with COVID-19 are in a state of high inflammation, increased stress and catabolism. Poor oral intake, which for 5 to 10 days before admission, is also common due to frequent coughing and breathlessness, dry mouth, and loss of taste and smell. Long stay in ICU, especially for intubated and ventilated patients, contributes to further malnutrition, loss of skeletal muscle mass, and disability. Though early and adequate enteral nutrition would be thought to mitigate these challenges and prevent gastrointestinal dysfunction, it has the potential for adverse reactions like abdominal distention, diarrhea, regurgitation, and overfeeding. Thus, the proper timing of optimal nutrients needed to meet the energy and protein requirements in critically ill patients with COVID-19 is debatable. We will conduct a subgroup analysis based on COVID status at baseline and assess the effect of protein intake on outcome. We will conduct a similar analysis to that of the main trial. Additional baseline laboratory tests, including ferritin, interleukin-6 (IL-6), lactate and procalcitonin, if available, will be compared between the two groups. In suspected or confirmed COVID patients, we will also compare the use of extracorporeal membrane oxygenation, inhaled nitric oxide, prone positioning, tracheostomy, intravenous immunoglobulins, and antiviral therapy.
2. ***The effect of protein supplementation according to nutritional risk:*** Malnutrition in critically ill patients is highly prevalent and associated with adverse clinical outcomes. Therefore, nutritional risk assessment is considered important to recognize high nutrition risk earlier and provide targeted nutritional therapy. However, there is a lack of consensus regarding the definition of nutritional risk. The Nutrition Risk in Critically ill (NUTRIC) score is the first nutritional risk assessment tool developed and validated specifically for ICU patients. The score includes age, APACHE II score, SOFA score, number of comorbidities, days from hospital admission to ICU admission, and IL-6. A modified version of the NUTRIC score, which excludes IL-6, has been validated in observational studies; the total score ranges from 0 to 9, with increasing scores indicating higher nutritional risk. Based on this score at baseline, we will conduct a pre-defined subgroup analysis on high vs. low NUTRIC patients. Other nutritional risk indicators that will be used are prealbumin (prealbumin ≤0.10 g/L considered as an indicator of severe nutritional risk, 0.11-0.15 g/L as mild to moderate risk, and >0.15 g/L as no risk), serum albumin (35 g/L considered as a cutoff value), baseline urine urea nitrogen (using the median of the cohort as a cutoff value), baseline nitrogen balance (positive versus negative balance), and SARC-F (1-3 versus ≥4).
3. ***The effect of protein supplementation* *across different BMI strata:*** With obesity increasing worldwide, there is also a rise in the prevalence of obesity in patients admitted to the ICU. Despite being associated with comorbid conditions, obesity has no independent effect on the outcome of critical illness other than increased ICU length of stay and increased severity of illness. On the other hand, underweight patients may have higher risk of mortality, possibly due to inadequate nutritional reserves to compensate for the stress of critical illness. Studies on the optimal dose and timing of enteral protein in critically ill patients according to their BMI are scarce. We will perform subgroup analyses stratified by BMI categories and evaluate the effect of protein intake on their outcomes. By definitions of the National Institutes of Health and World Health Organization, a person with a BMI <18.5 kg/m^2^ is underweight, 18.5 to 25 kg/m^2^ has normal weight, 25 to 29.9 kg/m^2^ is overweight, 30 to 39.9 kg/m^2^ is obese and ≥40 kg/m^2^ is morbidly obese.

- **Report generation**

**Figure legend**

**Figure 1:** CONSORT flow chart for the **REPLENISH** trial

**Supplemental Table Legends**

**Supplemental Table 1.** Baseline characteristics

**Supplemental Table 2.** Summary of interventions and co-interventions.

**Supplemental Table 3.** Primary outcome.

**Supplemental Table 4.** Secondary and safety outcomes.

**Supplemental Table 5.** Subgroup analyses.

**Supplemental Table 6.** Components of the SARC-F and EQ-5D-5L in the supplemental protein group and the control group

**Supplemental Table 7.** Prespecified subgroups.

**Supplemental Table 8.** Summary of Protocol Violations and Serious Adverse Events.

**Supplemental Table 9.** Additional information for COVID-19 patients.

**Planned figures in the final analysis:**

**Supplemental Figure 1.** Kaplan Meier Survival Curve for overall survival

**Supplemental Figure 2.** Serial parameters among patients in the supplemental protein group and control group.

1. Serial measurements of energy and protein
2. Serial measurements of glucose, insulin, nitrogen balance, prealbumin, ammonia, BUN, creatinine, 24-hour urine for urinary urea nitrogen.

**References**

- 1. Moher D, Hopewell S, Schulz KF, Montori V, Gotzsche PC, Devereaux PJ, Elbourne D, Egger M, Altman DG: CONSORT 2010 explanation and elaboration: updated guidelines for reporting parallel group randomised trials. BMJ 2010, 340:c869.
  2. Schulz KF, Altman DG, Moher D, Group C: CONSORT 2010 statement: updated guidelines for reporting parallel group randomised trials. BMJ 2010, 340:c332.
  3. International Conference on Harmonisation of Technical Requirements for Registration of Pharmaceuticals for Human Use: Good Clinical Practice (GCP) Guideline [<http://www.ich.org/fileadmin/Public_Web_Site/ICH_Products/Guidelines/Efficacy/E6/E6_R2__Step_4_2016_1109.pdf>]
  4. The International Council for Harmonisation of Technical Requirements for Pharmaceuticals for Human Use (ICH): STATISTICAL PRINCIPLES FOR CLINICAL TRIALS [<http://www.ich.org/fileadmin/Public_Web_Site/ICH_Products/Guidelines/Efficacy/E9/Step4/E9_Guideline.pdf>]
  5. International Conference on Harmonisation of Technical Requirements for Registration of Pharmaceuticals for Human Use: E3 - Structure and content of clinical study reports [<http://www.ich.org/fileadmin/Public_Web_Site/ICH_Products/Guidelines/Efficacy/E3/E3_Guideline.pdf>]
  6. Arabi YM, Aldawood AS, Haddad SH, et al. Permissive Underfeeding or Standard Enteral Feeding in Critically Ill Adults. N Engl J Med. 2015; 372: 2398-408.
  7. Krag M, Marker S, Perner A, et al. Pantoprazole in patients at risk for gastrointestinal bleeding in the ICU. New England Journal of Medicine. 2018; 379: 2199-208.
  8. Arabi YM, Al-Dorzi HM, Mehta S, et al. Association of protein intake with the outcomes of critically ill patients: a post hoc analysis of the PermiT trial. *Am J Clin Nutr*. 2018; 108: 988-96.
  9. Kahan BC, Harhay MO: Many multicenter trials had few events per center, requiring **analysis via random-effects models or GEEs**. *J Clin Epidemiol* 2015, **68**(12):1504-1511.
  10. Benjamini Y, Hochberg Y: **Controlling the false discovery rate: a practical and powerful approach to multiple testing**. *Journal of the Royal statistical society: series B (Methodological)* 1995, **57**(1):289-300.
  11. Kahan BC, Morris TP: **Reporting and analysis of trials using stratified randomisation in leading medical journals: review and reanalysis**. *BMJ* 2012, **345**:e5840.
